# Frequency and Mutation Patterns of Resistance in Patients with Chronic Hepatitis B Infection Treated with Nucleos(t)ide Analogs in Add-On and Switch Strategies

**DOI:** 10.5812/kowsar.1735143X.775

**Published:** 2011-10-01

**Authors:** Murat Sayan, Sila Cetin Akhan, Omer Senturk

**Affiliations:** 1 University of Kocaeli, Faculty of Medicine, Clinical Laboratory, Kocaeli, Turkey; 2 University of Kocaeli, Faculty of Medicine, Department of Clinical Bacteriology and Infectious Diseases, Kocaeli, Turkey; 3 University of Kocaeli, Faculty of Medicine, Department of Gastroenterology, Kocaeli, Turkey

**Keywords:** Hepatitis B Virus, Nucleoside, Mutation, Therapeutics

## Abstract

**Background:**

Treatment for chronic hepatitis B (CHB) has improved over the last 10 years mainly due to the development of effective oral antiviral agents [nucleoside/nucleotide analogs (NUCs)].

**Objectives:**

The aim of the present study is to identify the frequency and major patterns of resistance to the hepatitis B virus (HBV) in a Turkish population of CHB patients treated with NUCs using add-on and switch therapy strategies. Patients and Methods: The investigation involved a total of 194 patients (88 were treated using add-on therapy, and 106 were treated using switch therapy). We analyzed the HBV polymerase gene by amplification and direct sequencing procedures.

**Results:**

Primary drug-resistance mutations were detected in 84 patients (43%; 42 in add-on therapy, and 42 in switch therapy) taking lamivudine (LAM), 10 patients (5%; 6 in add-on therapy, and 4 in switch therapy) taking entecavir (ETV), and 16 patients (8%; 8 in add-on therapy, and 8 in switch therapy) taking adefovir (ADV). The most common LAM and ETV resistance mutations were rtM204I/V, rtL180M and rtT184A/I/S, respectively, while rtA181T/V and rtN236T substitutions were the most frequently observed ADV resistance mutations.

**Conclusions:**

Patients with CHB who developed NUC resistance were managed using 2 different rescue strategies. The frequency and mutation pattern of resistance were similar in patients treated with add-on and switch strategies. These findings may be helpful in the management of rescue strategies in LAM-resistant patients.

## 1. Background:

Treatment for chronic hepatitis B (CHB) has improved over the last 10 years mainly due to the development of effective oral antiviral agents (nucleoside/nucleotide analogs (NUCs)) [3]. NUCs used in hepatitis B virus (HBV) therapy belong to the following 3 classes:

L-nucleosides: lamivudine (LAM), telbivudine (LdT), and emtricitabine; Deoxyguanosine analogs: entecavir (ETV); Acyclic nucleoside phosphonates: adefovir dipivoxil (ADV) and tenofovir disoproxil fumarate (TDF).

Currently, LAM, ADV, ETV, TDF, and LdT have been licensed for the treatment of CHB [3][4].

Antiviral drug resistance now poses a major problem for the management of patients with CHB. Long-term therapy with NUCs, in particular, is associated with an increased risk of development of drug resistance [5][6]. In theory, resistance may be prevented if a sufficiently potent antiviral drug, or a combination of NUCs, is used such that viral replication and the ongoing selection of HBV quasispecies are prevented [5]. Mutations selected by treatment with NUCs can be split into 2 groups: those that cause resistances, sometimes leading to decreased viral fitness, and compensatory mutations, which partially or fully restore viral fitness [7]. The main mutation associated with LAM and L-nucleoside resistances is rtM204I/V, a mutation that occurs within the YMDD motif of the reverse transcriptase (rt) region of the polymerase [5]. Nine major mutation patterns associated with LAM resistance have been reported: (i) rtM204I/V + rtL180M; (ii) rtM204I; (iii) rtV173L + rtL180M + rtM204V; (iv) rtL80I + rtM204I; (v) rtQ215S + rtM204I/V ± rtL180M; (vi) rt169T + rtV173L + rtL180M + rtM204V; (vii) rtA181T; (viii) rtT184S + rtM204I/V ± rtL180M, and (ix) rtM204S + rtL180M. Some of these mutations can act as compensatory mutations, such as rtL80I/V and rtV173L [5]. LdT selects for mutations in the YMDD motif, and to date, only rtM204I (but not rtM204V) has been observed [3][8]. Two patterns of ETV resistance have been characterized, and they include the LAM-resistance mutation rtM204I/V plus an additional mutation of either rtT184G + rtS202I/C or rtM250V + rtI169T [5][9]. Similarly, the mechanism mediating ADV resistance has been characterized, with major resistance mutations located at rtN236T and/or rtA181T/V [6][10], and a number of other mutations found in 3 clusters within the rt region of the HBV polymerase [11]. In contrast, reports have shown that the TDF treatment induces little selection for viral resistance. For example, studies demonstrated that the rtA194T mutation in the HBV genome led to a decrease in HBV replication capacity with TDF treatment; however, additional and more long-term data investigating the effects of TDF treatment on HBV resistance are needed [3][7]. The complex patterns of mutations that can accumulate over time may affect the efficacy of subsequent treatments, suggesting that the identification of mutations will quickly become an important component in the management of patients with CHB [12].

NUC-resistant HBV variants can commonly be detected by direct sequencing of HBV DNA [13]. Sequence analysis is considered to be the gold standard for characterizing HBV DNA isolates [14]. However, the sensitivity of direct sequencing for the detection of minor variant populations is poor, and in general, these methods will detect mixtures of variant populations [15][16]. Sequencing a large number of clinical samples is time consuming, but it is suitable for screening large regions of the viral genome in order to detect potential compensatory mutations and new mutations [16].

In Turkey, where about 6500 individuals per year are infected with HBV, the virus is characterized by intermediate levels (2%–7%) of endemicity [17]. Moreover, studies conducted in Turkish patients having treated or untreated CHB infections indicated that HBV drug resistance is frequently mediated by rtM204V (YVDD variant) and rtM204I (YIDD variant) mutations, and rtM204S (YSDD variant) mutations with or without compensatory mutations such as rtV173L and rtL180M are also found, but occur much less frequently [18][19][20][21].

## 2. Objectives

In the present study, 2 different treatment strategies were applied for both hepatitis B e antigen (HBeAg)positive and HBeAg-negative CHB patients. The aim of the study is to determine the patterns and frequency of primary and compensatory mutations in patients undergoing long-term NUC treatment using add-on and switch therapy strategies.

## 3. Patients and Methods

### 3.1. Patients

This study was designed as a retrospective molecular study based on different rescue strategies implemented in the treatment of CHB patients with NUCs. All patients were consecutively enrolled. Molecular analysis was performed from March 2007 to November 2010 at the Kocaeli University Hospital. Patients with CHB were assigned to 2 groups: the first group (n = 88) was treated with NUCs using an add-on strategy (adding another drug effective against the drug-resistant mutant in patients from the Gastroenterology Department) after developing LAM resistance; the second group (n = 106) was treated with NUCs using a switch strategy (switching to a new antiviral agent after the development of resistance in patients from the Infectious Diseases Department). LAM (Zeffix, 100 mg/day; Glaxo Wellcome Laboratories, Middlesex, UK), ADV (Hepsera, 10 mg/day; Gilead Sciences Inc., Foster City, USA), ETV (Baraclude, 0.5 mg/day or 1 mg/day; Bristol-Myers Squibb Company, Princeton, USA), and TDF (Viread, 245 mg/day; Gilead Sciences Inc., Foster City, USA) were the oral anti-HBV drugs used in this study. Clinical and laboratory characteristics of patients in each group are shown in Table 1. Laboratory results revealed that all patients could be categorized as chronic HBV carriers according to the clinical practice guidelines of the European Association for the Study of the Liver (EASL) [4]. The histology activity index, an indicator of liver damage, was determined according to Knodell’s classification on a scale of 0 to 18 [22].

Blood samples were centrifuged immediately after collection, and the sera were separated, divided into aliquots, and then kept frozen at -20°C until testing. Serological markers of HBV were measured using commercially- available microparticle enzyme immunoassay kits (Axsym, Abbott Laboratories, IL, USA, and Elecsys, Roche Diagnostics, Mannheim, Germany).

### 3.2. DNA Isolation and Real-Time PCR

HBV DNA was isolated from serum samples with a biorobot workstation using magnetic-particle technology (NucliSENS-easyMAG, bioMérieux, Boxtel, Holland). HBV DNA was detected and quantified using a commercial real-time PCR assay and platform (Iontek Biyotechnology Inc., Istanbul, Turkey; and the iCycler iQ5 detection system, Bio-Rad Laboratories Inc., California, USA).

### 3.3. Sequencing of the HBV Polymerase Gene Region

Briefly, a pair of primers was designed (forward: 5’-TCGTGGTGGACTTCTCTCAATT- 3’ and reverse: 5’-CGTTGACAGACTTTCCAATCAAT- 3’) for amplification of the HBV polymerase region. PCR conditions were 95°C for 15 min, followed by 45 cycles consisting of 95°C for 45 s, 56°C for 45 s, and 72°C for 45 s. The final primer concentration was 0.3 μM, and the HBV amplicon size was 742 bp. All PCR products were purified using the High Pure PCR Product Purification Kit (Roche Diagnostics GmbH, Mannheim, Germany) and directly sequenced on ABI PRISM 310 Genetic Analyzer equipment using the DYEnamic ET Terminator Cycle Sequencing Kit (Amersham Pharmacia Biotech Inc., Piscataway, USA). For cycle sequencing, we used the following thermal protocol: 35 cycles consisting of 95°C for 20 s, 50°C for 25 s, and finally 60°C for 2 min. The reverse primer was used as the sequencing primer at a final concentration of 0.5 μM. Electropherogram-obtained sequences were assembled using Vector NTI v5.1 (InforMax, Invitrogen Life Science Software, Frederick, MD, USA).

### 3.4. Determination of HBV Genotype

HBV genotypes were determined using genotyping tools from the National Center for Biotechnology Information (NCBI, U.S. National Library of Medicine, Bethesda, USA, http://www.ncbi.nih.gov/projects/genotyping/ formpage.cgi.) to identify the genotype based on the viral nucleotide sequences. This genotyping tool works by using a BLAST search to compare a query sequence to a set of reference sequences for known genotypes [23]. The Genafor/Arevir geno2pheno drug resistance tool (Center of Advanced European Studies and Research, Bonn- Germany, http://www.geno2pheno.org/.) was used for HBV subgenotyping.

### 3.5. Determination of Polymerase and Surface Gene Mutations

Data accumulated by direct sequencing were analyzed either manually or using the geno2pheno tool. The Genafor/Arevir geno2pheno drug resistance tool for HBV is a database that is specifically designed for rapid computer- assisted virtual phenotyping of HBV, and accepts genome (nucleic acid) sequences as input. This geno2pheno program searches for homology between input sequences and other sequences already stored in its database, which includes relevant clinical data for drug resistances and surface gene mutations [3]. The tool searches for HBV drug resistance mutations in the rt domain of the polymerase at amino acid positions 80, 169, 173, 180, 181, 184, 194, 202, 204, 215, 233, 236, and 250. However, we also manually analyzed rt amino acid substitutions at positions 84, 85, 214, 237, and 238 [6]. The overlapping Sgene segment was evaluated using the geno2pheno tool for 5 amino acid substitutions at positions 137, 141, 144, 145, and 147 and using manual search at positions 121, 135, 139, 140, 142, 146, 148, 149, 151, 152, 153, 155, 156, and 157 [24].

### 3.6. Statistical Analysis

Age, HBV DNA load, alanine aminotransferase (ALT) levels, and aspartate aminotransferase (AST) levels were considered as the numerical values, while gender and HBeAg positivity represented the categorical variables. The significance of differences between 2 numeric variables was compared using Mann- Whitney U test. The significance of differences between 2 proportions was measured using Pearson Chi-square test. P values that were ≤0.05 were considered statistically significant. Statistical analyses were conducted using SPSS software version 13.0.0 (SPSS Inc., IL, USA) for Windows.

## 4. Result

Demographic data and clinical features for each of the study groups are summarized in Table 1. Mutations inthe HBV polymerase gene known to confer resistance to NUCs were found in 122 out of 194 patients (63%). Of these 122 patients, 68 (56%) were in the add-on therapy group and 54 (45%) were in the switch therapy group. Four different LAM resistance patterns were identified in 84 out of 194 patients (43%): baseline primary mutation (rtM204I/V, n = 26), primary mutation with the compensatory mutations rtL80I/V (n = 16) or rtL180M (n = 24), triple mutant (rtM204I/V + rtV173L + rtL180M or rtA194G, n = 12), and the single mutation rtL180M (n = 6). The most frequent mutations detected in response to LAM treatment were rtM204I/V and rtL180M Table 2. LAM-resistance mutations (included ETV resistance mutations) were identified in 48 out of 88 patients (55%) in the addon therapy group (median LAM therapy duration, 28.7 months, with an LAM + ADV combination therapy duration of 23.3 months) and in 46 out of 106 patients (43%) in the switch therapy group (median therapy duration, 24.8 months). This difference was not statistically significant (P > 0.05) Table 1 and Table 2. LdT-resistance patterns were identified in 60 out of 194 patients (31%), and there were no significant differences in the frequencies of these mutations between the add-on and switch therapy groups (P > 0.05). The observed patterns of LdT resistance included baseline primary mutations (rtM204I, n = 24) associated with the compensatory mutations rtL80I/V or rtL180M (n = 28) and triple mutants (rtM204I + rtV173L + rtL180M or rtL80I/V, n = 8; Table 1).

**Table 1:** Demographic Data and Clinical Features of Patients in Each Study Group

	**aAdd-on Strategy, (n = 88) **	**Switch Strategy, (n = 106)**
Male, No. (%)	66 (75)	70 (66)
Age, median y (range)	45 (13–68)	38 (16–61)
HBeAg positive, No. (%)	30 (34)	36 (33.9)
ALT b, median U/L (range)	68 (16–537)	84 (12–1082)
AST b, median U/L (range)	61 (14–709)	55 (13–579)
HBV DNA, median log10 IU/mL (range)	4.4 (2.0–6.0)	3.8 (2.0–6.1)
HBV subgenotype D, No. (%)		
D1	74 (84)	82 (77)
D2	12 (14)	12 (11)
D3	2 (2)	10 (10)
D4	0 (0)	2 (2)
History of chronic hepatitis B infection		
Patients in the immune-tolerant phase	24	22
Patients in the immune-reactive phase	6	14
HBeAg-negative patients	58	70
Biopsy status		
Patients with Knodell fibrosis scores	40	72
Patients without biopsy	48	34
Therapy status c		
LAM b to LAM + ADV b	74	-
ADV to ADV + ETV b	14	-
LAM to ADV + ETV	-	12
LAM to ADV + TDF b	-	10
LAM to ETV	-	24
ADV to ETV	-	30
Treatment duration, median mon (range)		
LAM	28.7 (3–60)	24.8 (2–126)
LAM + ADV	23.3 (6–48)	-
ADV	-	13.1 (3–36)
ADV + ETV	16 (6–22)	-
ETV	-	11.4 (6–24)

^a^ Serological markers of all patients were found to be negative for hepatitis C virus and hepatitis D virus.

^b^ Abbreviations: ADV, adefovir dipivoxil; ALT, alanine aminotransferase; AST, aspartate aminotransferase; ETV, entecavir; LAM, lamivudine; TDF, tenofovir disoproxil fumarate.

^c^ The combination of NUCs used was selected according to the emergence of drug resistance (primary or compensatory resistance) or clinical and/or virological breakthrough.

**Table 2:** Determination of NUC-Associated Mutational Patterns in Different Therapy Strategies

**Genotypic Resistance Mutation(n = 194)**	**Patients, No. (%)**	**Add-On Strategy(n = 88)**	**SwitchStrategy(n = 106)**	***P *value b**
No mutations	72 (37%)	20	52	< 0.001
Primary mutations				
LAM a	84 (43%)	42	42	
rtM204I/V	26	14	12	
rtM204I/V + rtL80I/V	16	16	8	
rtM204I/V + rtL180M	24	14	10	
rtM204I/V + rtV173L + rtL180M or rtA194G	12	4	8	
rtL180M	6	2	4	
ETV a	10 (5%)	6	4	> 0.05
rtM204I/V + rtL180M + rtT184A/I/S	8	4	4	
rtM204V + rtL180M + rtS202C	2	2	0	
ADV a	16 (8%)	8	8	> 0.05
rtA181T/V	6	4	2	
rtN236T	8	4	4	
rtA181T + rtN236T	2	0	2	
LdT a, c	60 (31%)	30	30	> 0.05
rtM204I	24	14	10	
rtM204I + rtL180M or rtL80I/V	28	12	16	
rtM204I + rtL180M + rtV173L or rtL80I/V	8	4	4	
Compensatory mutations	12 (6%)	12	0	
rtQ215H/P/S ± rtV214A/P	12	12	0	< 0.01

^a^ Abbreviations: ADV, adefovir dipivoxil; ETV, entecavir; LAM, lamivudine; LdT, telbivudine.

^b^ Based on the 2 proportion significance tests.

^c^ LdT was not used in either of the therapy strategies. Nevertheless, the patterns and rate of drug resistance to LdT are shown as additional data.

The ADV-associated mutations rtA181T/V, rtN236T, and rtA181T + rtN236T were detected in 6, 8, and 2 patients, respectively (for a total of 16 out of 194 patients, or 8%). Primary ADV-resistance mutations (i.e. N236T) were most frequently detected. ADV-resistance mutations were identified in 8 out of 88 patients (9%) in the add-on therapy group (median ADV therapy duration, LAM + ADV: 23.3, ADV + ETV: 19.5 months) and in 8 out of 106 patients (7.5%) in the switch group (median therapy duration, 13.1 months). This difference was not statistically significant (P > 0.05; Table 1 and Table 2. Single compensatory mutations were not detected in the switch therapy group; however, rtQ215H/P/S ± rtV214A/P mutations were found in 12 out of 88 patients (14%) in the add-on therapy group, and this difference was significant (P < 0.01).

The  ETV-resistance  mutation rtM204I/V  +  rtL180M + rtT184A/I/S or rtS202C was found in 10 out of 194 patients (5%). Of these 10 patients, 6 out of 88 (7%) were in the add- on therapy group (median ETV therapy duration as ADV + ETV combination, 19.5 months) and 4 out of 106 (4%) were in the  switch therapy group (median ETV therapy duration, 11.4 months). There was no significant difference in ETV drug resistance mutations between the 2 groups (P > 0.05;Table 1 and Table 2).

Mutations conferring resistance to  TDF were  not  detected. In 1 patient in the add-on therapy group, a multi- drug-resistant HBV strain was detected during combination  therapy with  LAM and  ADV. Eight patients also had changes in the  amino acid  sequence of the  overlapped S-gene  segment. Two  patients in  the   add-on therapy group had sC137G amino acid substitutions (with rtL80M + rtM204I  mutations), 2 patients in the  switch therapy group had  sG145R mutations (with  rtN236T mutations), and  4 patients in the  switch therapy group had  sD144E mutations (1 patient with  rtL80M + rtM204V mutations and 3 patients with rtQ215S mutations).

Direct sequencing results revealed that all patients had HBV genotype D. Subgenotype D1 was identified as the HBV genotype in 74 out  of 88 patients (84%) and  82 out of 106 patients (77%) in the  add-on and  switch therapygroups, respectively. Of the remaining patients, 12 out of 88 (14%) and  12 out  of 106 (11%) were  categorized as sub- genotype D2, while 2 out of 88 (2%) and 10 out of 106 (10%) were  categorized as subgenotype D3, in the  add-on and switch therapy groups, respectively. Subgenotype D4 was detected only in 2 out  of 106 patients (2%) in the  switch therapy group Table 1.

Patients in the  add-on and  switch therapy groups had median HBV viral loads  of 4.4 log10  (2.0–6.0) and  3.8log10 (2.0–6.1) IU/mL, median ALT levels of 68 (16–537) U/L and 84 (12–1082) U/L, and median AST levels of 61 (14–709) and 55 (13–579) U/L, respectively Table 1. Differences in HBV viral load and ALT/AST levels in patients in the add-on and switch therapy groups were not significant according to Mann-Whitney U test (P = 0.310, 0.503, and  0.794, respectively).  For patients in  the  add-on and  switch therapy groups, the  median age was 45 years  (range, 13–68) and 38 years (range, 16–61), respectively, and  the number and percentage of male  patients was 66 (75%) and  70 (66%), respectively.  Further,  HBeAg positivity was  detected in 30  patients (34%) and  36 patients (33.9%), respectively (Table 1). Differences in age, gender, and HBeAg positivity were not significantly between the 2 groups according to Pearson Chi-square test(P = 0.412, P = 0.554, and P = 0.601, respectively). Furthermore, demographic data  and  clinical features, including age, gender, HBeAg positivity, HBV DNA load, ALT levels, and AST levels were not found to be related to the detection of drug resistance mutations.

Knodell fibrosis scores  were assigned to 40 patients in the add-on group and 72 patients in the switch group: 19% and 15% were scored as 1; 67% and 75% were scored as 2; 11% and  10% were scored as 3; and  3% and  0% were scored as 5, respectively. None of these scores  correlated with  the presence of mutations.

## 5. Discussion

The long-term use of monotherapy drugs in CHB, and LAM in particular, is frequently associated with  the  development of  drug resistance. Ideally,  antiviral drugs used   in  combination should be  carefully chosen to have different mechanism or sites  of action and  should act in an additive or synergistic fashion [5][25]. According  to  a recently published study,  time-limited add-on strategies do  not  provide benefits over  switch strategies  with   respect to  the   emergence of  ADV-resistant mutants in LAM-resistant CHB patients [26]. In contrast, add-on ADV therapy was found to be more effective  and longer lasting than ETV as a rescue therapy in patients with  LAM-resistant  mutations who  required long-term antiviral treatment [27]. According to EASL clinical and practice guidelines for cases  of drug resistance, an  appropriate rescue therapy should be  initiated with  the most effective  NUCs and  with  minimal risk of inducing multiple drug-resistant strains. Therefore, the only effective strategy is the  addition of a second drug that does not  display cross-resistance [4]. The results of this  study demonstrated that add-on and  switch therapy strategies  using NUCs to  treat CHB conferred similar major drug-resistance patterns at approximately the  same  frequency, with  the  exception of compensatory mutations such as rtQ215S. Major drug-resistance patterns resulting from  LAM and  LdT treatment occurred more frequently than ADV- or ETV-resistance patterns. Resistance to LAM and  LdT may develop when using these agents in long- term treatment (median treatment duration with  LAM: 28.7 months in the add-on group and 24.8 months in the switch group; Table 1). In fact, studies have  shown that the  frequency of viral resistance progressively increases from  10%–27% at the  initial diagnosis to 37%–48% by the end  of the  second year of LAM monotherapy [3]. In our study,  the  rtM204V  mutation was  detected as a single mutation in only 1 patient under monotherapy. This mutation is usually not  detected as a single mutation, but instead is usually found in  combination with  rtL180M [5]. Replication defects resulting from  NUC treatment can be partly compensated for by selection for compensatory  mutations [7][28]. The rtL180M mutation was the most common compensatory mutation  in  our  study. In vitro  studies have  demonstrated that this  mutation alone is insufficient to  result in  LAM resistance; however, this  mutation augments both viral replication and LAM resistance in  the  context of rtM204I/V mutations. The rtV173L HBV mutation occurs in 9% of LAM-resistant patients [5] and  serves  to  further increase the  replication   capacity of  poorly replicating LAM-resistant  HBV [2]. Mutation at codon rtL80I/V may  also be an alternative compensatory mutation to rtL180M in HBV genomes encoding the rtM204I  mutation, and  this mutation may act in a manner similar to rtQ215S. In the  current study, mutations at rtQ215S were  only  observed in the  add-on therapy group in 12 patients (14%), while previous studies have described the rtQ215S mutation as a polymorphism that is detected in ~12% of patients treated with  LAM [5]. Substitutions at  rtQ215 also  occur  during ADV therapy [6]. However,  Olyaee et al. demonstrated no association between rtQ215 mutations and  clinical complications in patients infected with  HBV genotype D [29]. Accordingly, in our  previously published report, we found that rtQ215A/H/P/S substitutions could be detected as naturally occurring mutations in treatment-naïve patients with CHB [30]. Furthermore, rtQ215H/P/S substitutions occur as  dominant  compensatory mutations  in  treatment-naïve hemodialysis patients infected with HBV genotype D (unpublished data).  However,  in vitro  studies suggest that rtQ215 substitutions impair neither viral replication efficiency  nor  susceptibility to LAM or ADV [29]. LdT, another nucleoside analog, also selects for mutations in the YMDD motif,  similar to mutations conferring resistance to LAM, and  is not  expected to be effective  in LAM-resistant patients. Resistance to LdT also begins to emerge in the  first  year  of treatment and  progressively increases during the  second year of therapy [3]. LdT was not  used in  either therapy strategy implemented in  this  study. Nevertheless, we included the frequency and patterns of LdT drug resistance in Table 2, according to EASL clinical and practice guidelines [4].

The rtA181T HBV mutation is a major mutation pattern associated with  LAM resistance, but  selected for during ADV  treatment and  during the  development of  resistance to combination (ADV + LAM) therapy in the absence of mutations at rtM204I/V [5]. In this study, rtA181T mutations were detected in 6 patients who did not  also carry the rtM204I/V mutation. ADV drug resistance detected in this  study was found to be associated with  mutations at rtN236T and  rtA181T/V. Further, rtQ215S and  rtV214A mutations were compensatory mutations arising from  ADV treatment [10]. However, while  the rtN236T substitution does  not  significantly affect sensitivity to LAM, rtA181T/V and rtQ215S/rtV214A mutations confer partial cross-resistance to LAM [31].

Resistance to  ETV  is  associated with  2 different mutation profiles, both of  which  include  LAM-resistance mutations [5][9][32]. High  levels  of  ETV  resistance resulted from  dual  rtM250V and  rtI169T mutations, and we detected another profile,  the  rtM204I/V + rtL180M + rtT184A/I/S or rtS202C triple mutation, as an ETV-resistant mutation in both NUC therapy strategies. On the  other hand, 24 patients receiving LAM treatment in the switch therapy group were switched to ETV therapy at 1 mg daily (Table 2), and  while these patients had no LAM-resistance mutations, they  were  refractory to LAM therapy. In this group of LAM-refractory patients, the  emergence of ETV resistance occurred more frequently than was the  case with  NUC treatment-naïve patients [33]. ETV and  TDF are both potent HBV inhibitors and have a high barrier to resistance. Thus, they  can be confidently used  as first-line monotherapies [4].

HBV strains that are  resistant to  at  least  2 anti-HBV agents from   different NUC subclasses without  cross- resistance  profiles are  defined as  multidrug-resistant strains [3]. These  multidrug-resistant strains are  more likely  to develop additional mutations with  sequential therapy [34][35][36]. In the  current study,  we found 1 patient with  dual  ADV (rtN236T + rtQ215H) and  LAM (rtM204V + rtL180M + rtV173L) resistance, detected in different serum samples from the same  patient. Recently, the emergence of the  first  multidrug-resistant HBV strain arising from sequential oral anti-HBV therapies was documented [35]. Nevertheless, there is limited in vivo data  demonstrating resistance to multiple NUCs [3].

Because of overlapping open reading frames of the HBV polymerase and  the  HBsAg, drug resistant mutations in the  HBV polymerase can have a direct impact on the nature of the  HBsAg and  its function [28][37]. Mutations in and  around the major neutralization domain of HBV, known as the  ‘a’ determinant, may  result in decreased affinity  of the  HBsAg to  anti-HBs  and  cause  diagnostic problems and/or failure to prevent infections by vaccination  or HB immunoglobulin [7][38]. Studies  have shown that LAM-resistant HBV (harboring the rtV173L + rtL180M + rtM204V triple mutation) has significantly reduced anti-HBs binding capacity due to changes in the HBsAg [37]. Both our previous studies and the present study demonstrate that the overlapping S-gene segment (sC137, sG145, and  sD144) was affected by genotypic resistance associated with NUC treatment [39]. Mutant sG145R is the most frequently reported modification and is a known vaccine escape  mutant [7][40]. It appears to seriously impair the performance of many commercial tests  and  is responsible  for false-negative results [37]. However,  the  Axsym and Elecsys tests  used in this study successfully detected this mutation.

The occurrence of NUC-resistant HBV variants can com- monly be detected by direct sequencing of HBV DNA [41]. This  method is  required for  the  identification of  any novel  mutation  within the  target gene  [1][12]. Further- more, while direct sequencing is a time-consuming task when dealing with  a large  numbers of clinical samples, it is suitable for the screening of a large region of the viral genome (in this  case, the HBV polymerase gene). The geno2pheno program is a useful tool  that facilitates a simple and  rapid analysis of mutations associated with NUC resistance in large  regions of the  HBV genome. In this  study,  we used  both manual analysis of sequence mutations and the geno2pheno drug resistance tool. We found similar results when analyzing our data  manually and  using the geno2pheno program, with the exception of 1 patient with ETV drug resistance (rtM204V + rtL180M + rtT184S). DNA chromatograms obtained from  this  patient show  2 peaks  for the  same  nucleotide signal (both a wild- and  mutant-type peak).  Because the  base calling algorithm used by drug resistance tools selects  the most prominent signal to define each nucleotide, both manual analysis and  the  use of drug resistance tools,  such  as geno2pheno, should be used  together to interpret drug resistance mutations.

Several studies have  shown that HBV genotype D rep- resents almost all isolates from  the  Turkish HBV patient population [33][39][42][43]. However,  we recently docu- mented a case of HBV genotype A in Turkish patient {%44). The present study demonstrated that HBV genotype D is still dominant among Turkish CHB-infected patients. Be- cause  of the  dominance of genotype D, it is difficult to evaluate NUC resistance in relation to the different genotypes  of HBV. A limited number of reports have demonstrated the  relationship between HBV genotype and  the response to antiviral therapy with LAM [15]. Zollner et al. reported that the  mutational pattern during the  selection  of LAM-resistant HBV strains differs  between genotypes A and D. However, EASL clinical practice guidelines have  not  yet  supported any  relationship between the HBV genotype and  response to NUC therapy [4]. To date, there is little  data  describing the subgenotyping of HBV in Turkish patients. In the  present study,  HBV pol  gene sequences isolated from  Turkish patients revealed that subgenotype D1 constitutes the  majority of genotype D circulating in  Turkey. We also  demonstrated the  presence of subgenotypes D2, D3, and  D4; however, based onpol gene sequencing, HBV subgenotype D1 was predominant (84%) and  subgenotypes D2, D3, and  D4 were found in only 10%, 5%, and  0.2% of Turkish patients with  CHB (n = 442), respectively [45]. In contrast, other groups have shown that subgenotype D2 is the predominant HBV sub- genotype in Turkish patients [46][47][48]. While  both these studies use pre-S gene  amplification along with  restriction   fragment length polymorphism techniques, the discrepancies in the reported results are probably due to differences in methodology.

In conclusion, CHB is a disease that can  develop progressive  resistance to  NUCs by  mutations in  the  HBV polymerase gene.  Therefore, it is necessary to use effective  therapeutic strategies to  manage drug resistance. However, we did not detect a significant difference in the emergence of major drug resistance patterns and  their frequencies when add-on and   switch strategies  were implemented. These findings may prove  to be useful in the  management of  rescue strategies in  LAM-resistant patients.

## References

[1] Mittal A, Airon RK, Magu S, Rattan KN, Ratan SK (2004). Associated anomalies with anorectal malformation (ARM). Indian J Pediatr.

[2] Delaney WEt, Yang H, Westland CE, Das K, Arnold E, Gibbs CS, Miller MD, Xiong S (2003). The hepatitis B virus polymerase mutation rtV173L is selected during lamivudine therapy and enhances viral replication in vitro. J Virol.

[3] Papatheodoridis GV, Deutsch M (2008). Resistance issues in treating chronic hepatitis B. Future Microbiol.

[4] (2009). EASL Clinical Practice Guidelines: management of chronic hepatitis B. J Hepatol.

[5] Bartholomeusz A, Locarnini SA (2006). Antiviral drug resistance: clinical consequences and molecular aspects. Semin Liver Dis.

[6] Shaw T, Bartholomeusz A, Locarnini S (2006). HBV drug resistance: mechanisms, detection and interpretation. J Hepatol.

[7] Sheldon J, Rodes B, Zoulim F, Bartholomeusz A, Soriano V (2006). Mutations affecting the replication capacity of the hepatitis B virus. J Viral Hepat.

[8] Lai CL, Leung N, Teo EK, Tong M, Wong F, Hann HW, Han S, Poynard T, Myers M, Chao G, Lloyd D, Brown NA, Telbivudine Phase II Investigator Group (2005). A 1-year trial of telbivudine, lamivudine, and the combination in patients with hepatitis B e antigen-positive chronic hepatitis B. Gastroenterology.

[9] Tenney DJ, Levine SM, Rose RE, Walsh AW, Weinheimer SP, Discotto L, Plym M, Pokornowski K, Yu CF, Angus P, Ayres A, Bartholomeusz A, Sievert W, Thompson G, Warner N, Locarnini S, Colonno RJ (2004). Clinical emergence of entecavir-resistant hepatitis B virus requires additional substitutions in virus already resistant to Lamivudine.. Antimicrob Agents Chemother.

[10] Angus P, Vaughan R, Xiong S, Yang H, Delaney W, Gibbs C, Brosgart C, Colledge D, Edwards R, Ayres A, Bartholomeusz A, Locarnini S (2003). Resistance to adefovir dipivoxil therapy associated with the selection of a novel mutation in the HBV polymerase. Gastroenterology.

[11] Bartholomeusz A, Locarnini SA, Ayres A, Thompson G, Sozzi V, Angus P, Sievert W, Sasadeusz J, Chalmers D, Cooper M (2004). Molecular modelling of hepatitis B virus polymerase and adefovir resistance identifies three clusters of mutations. Hepatology.

[12] Yuen LK, Ayres A, Littlejohn M, Colledge D, Edgely A, Maskill WJ, Locarnini SA, Bartholomeusz A (2007). SeqHepB: a sequence analysis program and relational database system for chronic hepatitis B. Antiviral Res.

[13] Jardi R, Rodriguez-Frias F, Schaper M, Ruiz G, Elefsiniotis I, Esteban R, Buti M (2007). Hepatitis B virus polymerase variants associated with entecavir drug resistance in treatment-naive patients. J Viral Hepat.

[14] Sertoz RY, Erensoy S, Pas S, Akarca US, Ozgenc F, Yamazhan T, Ozacar T, Niesters HG (2005). Comparison of sequence analysis and INNO-LiPA HBV DR line probe assay in patients with chronic hepatitis B. J Chemother.

[15] Niesters HG, Pas S, de Man RA (2005). Detection of hepatitis B virus genotypes and mutants: current status. J Clin Virol.

[16] Bartholomeusz A, Locarnini S (2006). Hepatitis B virus mutations associated with antiviral therapy. J Med Virol.

[17] Shepard CW, Simard EP, Finelli L, Fiore AE, Bell BP (2006). Hepatitis B virus infection: epidemiology and vaccination. Epidemiol Rev.

[18] Akarsu M, Sengonul A, Tankurt E, Sayiner AA, Topalak O, Akpinar H, Abacioglu YH (2006). YMDD motif variants in inactive hepatitis B carriers detected by Inno-Lipa HBV DR assay. J Gastroenterol Hepatol.

[19] Akyildiz M, Gunsar F, Ersoz G, Karasu Z, Ilter T, Batur Y, Akarca U (2007). Adefovir dipivoxil alone or in combination with lamivudine for three months in patients with lamivudine resistant compensated chronic hepatitis B. Dig Dis Sci.

[20] Tuncbilek S, Kose S, Elaldi A, Akman S (2008). Lamivudine resistance in untreated chronic hepatitis B patients in Turkey. Turk J Gastroenterol.

[21] Bozdayi AM, Uzunalimoğlu O, Türkyilmaz AR, Aslan N, Sezgin O, Sahin T, Bozdayi G, Cinar K, Pai SB, Pai R, Bozkaya H, Karayalçin S, Yurdaydin C, Schinazi RF (2003). YSDD: a novel mutation in HBV DNA polymerase confers clinical resistance to lamivudine. J Viral Hepat.

[22] Knodell RG, Ishak KG, Black WC, Chen TS, Craig R, Kaplowitz N, Kiernan TW, Wollman J (1981). Formulation and application of a numerical scoring system for assessing histological activity in asymptomatic chronic active hepatitis. Hepatology.

[23] Rozanov M, Plikat U, Chappey C, Kochergin A, Tatusova T (2004). A web-based genotyping resource for viral sequences. Nucleic Acids Res.

[24] Avellon A, Echevarria JM (2006). Frequency of hepatitis B virus 'a' determinant variants in unselected Spanish chronic carriers. J Med Virol.

[25] Marcellin P, Asselah T, Boyer N (2005). Treatment of chronic hepatitis B. J Viral Hepat.

[26] Idilman R, Kaymakoglu S, Oguz Onder F, Ahishali E, Bektas M, Cinar K, Pinarbasi B, Karayalcin S, Badur S, Cakaloglu Y, Mithat Bozdayi A, Bozkaya H, Okten A, Yurdaydin C (2009). A short course of add-on adefovir dipivoxil treatment in lamivudine-resistant chronic hepatitis B patients. J Viral Hepat.

[27] Chung GE, Kim W, Lee KL, Hwang SY, Lee JH, Kim HY, Jung YJ, Kim D, Jeong JB, Kim BG, Kim YJ, Yoon JH, Lee HS (2011). Add-on adefovir is superior to a switch to entecavir as rescue therapy for Lamivudine-resistant chronic hepatitis B. Dig Dis Sci.

[28] Warner N, Locarnini S, Kuiper M, Bartholomeusz A, Ayres A, Yuen L, Shaw T (2007). The L80I substitution in the reverse transcriptase domain of the hepatitis B virus polymerase is associated with lamivudine resistance and enhanced viral replication in vitro. Antimicrob Agents Chemother.

[29] Amini-Bavil-Olyaee S, Herbers U, Mohebbi SR, Sabahi F, Zali MR, Luedde T, Trautwein C, Tacke F (2009). Prevalence, viral replication efficiency and antiviral drug susceptibility of rtQ215 polymerase mutations within the hepatitis B virus genome. J Hepatol.

[30] Sayan M, Akhan SC, Meric M (2010). Naturally occurring amino-acid substitutions to nucleos(t)ide analogues in treatment naive Turkish patients with chronic hepatitis B. J Viral Hepat.

[31] Locarnini S, Mason WS (2006). Cellular and virological mechanisms of HBV drug resistance. J Hepatol.

[32] Sayan M (2010). Molecular Diagnosis of Entecavir Resistance. Hepat mon.

[33] Sherman M, Yurdaydin C, Simsek H, Silva M, Liaw YF, Rustgi VK, Sette H, Tsai N, Tenney DJ, Vaughan J, Kreter B, Hindes R, AI463026 Benefits of Entecavir for Hepatitis B Liver Disease (BEHoLD) Study Group (2008). Entecavir therapy for lamivudine-refractory chronic hepatitis B: improved virologic, biochemical, and serology outcomes through 96 weeks. Hepatology.

[34] Karatayli E, Karayalçin S, Karaaslan H, Kayhan H, Türkyilmaz AR, Sahin F, Yurdaydin C, Bozdayi AM (2007). A novel mutation pattern emerging during lamivudine treatment shows cross-resistance to adefovir dipivoxil treatment. Antivir Ther.

[35] Villet S, Pichoud C, Villeneuve JP, Trepo C, Zoulim F (2006). Selection of a multiple drug-resistant hepatitis B virus strain in a liver-transplanted patient. Gastroenterology.

[36] Sayan M, Hulagu S, Karatayli SC (2010). Multidrug-Resistant Hepatitis B Virus Strain in a Chronic Turkish Patient. Hepat Mon.

[37] Torresi J (2002). The virological and clinical significance of mutations in the overlapping envelope and polymerase genes of hepatitis B virus. J Clin Virol.

[38] Weber B (2005). Genetic variability of the S gene of hepatitis B virus: clinical and diagnostic impact. J Clin Virol.

[39] Sayan M, Senturk O, Akhan SC, Hulagu S, Cekmen MB (2010). Monitoring of hepatitis B virus surface antigen escape mutations and concomitantly nucleos(t)ide analog resistance mutations in Turkish patients with chronic hepatitis B. Int J Infect Dis.

[40] Locarnini SA (1998). Hepatitis B virus surface antigen and polymerase gene variants: potential virological and clinical significance. Hepatology.

[41] Pei F, Ning JY, You JF, Yang JP, Zheng J (2005). YMDD variants of HBV DNA polymerase gene: rapid detection and clinicopathological analysis with long-term lamivudine therapy after liver transplantation. World J Gastroenterol.

[42] Bozdayi AM, Aslan N, Bozdayi G, Turkyilmaz AR, Sengezer T, Wend U, Erkan O, Aydemir F, Zakirhodjaev S, Orucov S, Bozkaya H, Gerlich W, Karayalçin S, Yurdaydin C, Uzunalimoğlu O (2004). Molecular epidemiology of hepatitis B, C and D viruses in Turkish patients. Arch Virol.

[43] Sayan M, Hulagu S, Akhan SC, Senturk O, Meric M, Cekmen M (2009). [Entecavir resistance in entecavir naive lamivudine treated chronic hepatitis B patients]. Mikrobiyol Bul.

[44] Sayan M, Akhan SC, Bozdayi M (2010). Genotype A2_adw2 Strain of Hepatitis B Virus in Turkey: A Case Report. Hepat Mon.

[45] Sayan M, Akhan SC (2011). Antiviral drug-associated potential vaccine-escape hepatitis B virus mutants in Turkish patients with chronic hepatitis B. Int J Infect Dis.

[46] Leblebicioglu H, Eroglu C (2004). Acute hepatitis B virus infection in Turkey: epidemiology and genotype distribution. Clin Microbiol Infect.

[47] Ozdemir FT, Duman D, Ertem D, Avşar E, Eren F, Ozdoğan O, Kalayci C, Aslan N, Bozdayi AM, Tözün N (2005). Determination of hepatitis B genotypes in patients with chronic hepatitis B virus infection in Turkey. Gastroenterol.

[48] Sunbul M, Leblebicioglu H (2005). Distribution of hepatitis B virus genotypes in patients with chronic hepatitis B in Turkey. World J Gastroenterol.

